# Impact of Electron Beam Treatment and Storage Duration on Microbial Stability and Phytochemical Integrity in Hemp Flowers

**DOI:** 10.3390/molecules30173601

**Published:** 2025-09-03

**Authors:** Fernando D. Goffman, Dániel Á. Carrera, Diogo A. R. S. Latino, Christelle Cronje, Leron Katsir

**Affiliations:** 1 Seedcraft S.L., Paraje Casa de las Cebollas 1, 30510 Yecla, Región de Murcia, Spain; fernando.goffman@seedcraft.org; 2 Puregene AG, Etzmatt 273, 4314 Zeiningen, Switzerland; carrera@pureeurope.eu (D.Á.C.); cronje@pureeurope.eu (C.C.); 3 Independent Researcher, 6300 Zug, Switzerland; diogolatino@gmail.com

**Keywords:** cannabinoids, cannabis, decontamination, electron beam irradiation, hemp, microbial safety, preservation, terpenes

## Abstract

This study compared the effects of storage time and electron beam (EB) irradiation on microbial counts and chemical stability of dried flowers from two hemp cultivars over 12 weeks. Cannabinoid and terpene content, as well as microbial load, were evaluated at 0, 4, 8, and 12 weeks in EB-irradiated and non-irradiated samples. Microbial count in non-irradiated flowers reached up to 4.1 × 10^6^ colony-forming units (CFU)/g; EB irradiation reduced these levels to <10^2^ CFU/g. Cannabinoid contents were unaffected by EB irradiation and remained stable throughout storage. Terpene content decreased by 8.4% immediately after irradiation, followed by further declines during storage, reaching 22.3% and 24.0% average losses in non-irradiated and EB-irradiated samples after 12 weeks, respectively. EB irradiation caused a higher decrease in monoterpenes (10.8%) than in sesquiterpenes (2.5%). These findings confirm that EB irradiation is an effective sterilization method for hemp flowers that preserves chemical integrity. Storage time also significantly reduced microbial loads in non-irradiated samples; TAMC in cultivar B declined from 20,728 CFU/g to <LOQ (100 CFU/g), and TYMC decreased 16-fold. Cultivar A exhibited a sharp initial TAMC reduction followed by fluctuations and TYMC levels that were 7-fold lower by Week 12, reflecting natural microbial decay during hemp flower storage.

## 1. Introduction

Cannabis (*Cannabis sativa* L.) one of the oldest cultivated plants [1] is valued for its diverse array of applications, including fiber, textiles, fuel, therapeutics, and pharmaceuticals [2,3,4]. Owing to the history of global prohibition, cannabis has been broadly classified as either hemp, which is characterized by low Δ9-tetrahydrocannabinol (THC) content and permitted for cultivation, or cannabis plants with higher THC levels that remain strictly regulated. The legal classification of hemp varies by jurisdiction. In the United States, hemp is defined as containing no more than 0.3% THC by dry weight [5], whereas in Switzerland, the allowable THC limit can be as high as 1% [6]. Cannabis flower is the major source of the plant’s diverse array of phytochemicals, including cannabinoids, terpenes, and phenolic compounds, which contribute to its pharmacological and therapeutic properties. Hemp, in particular, serves as a rich source of cannabidiol (CBD), a non-psychoactive cannabinoid that is less abundant in high-THC cultivars [7,8,9]. Similar to the broad medical applications for high-THC medical cannabis, such as in treatments for chronic pain, inflammatory diseases, and chemotherapy-induced nausea [10], hemp flower and CBD-rich extracts have been recognized for their therapeutic potential [11]. Many studies have demonstrated the potential of CBD in alleviating pain [12,13,14,15] and anxiety [16], and in management of seizures associated with epilepsy [17]. Hemp and medical cannabis are primarily consumed by inhalation, either through vaporization or smoking of the dried flower [18]. However, during cultivation, processing, and packaging, hemp can become contaminated by pathogenic fungi such as yeasts and molds [19], which pose safety concerns, especially when inhaled [20,21,22,23]. Previous studies have linked fungal contamination in cannabis products to serious health outcomes [24,25,26,27,28], underscoring the need for improved microbial safety standards for hemp flower.

For medicinal cannabis, consumer safety concerns have led to regulations in Europe, Canada, and certain U.S. jurisdictions that mandate microbial load testing. Under Dutch regulations, medicinal cannabis must contain <100 colony-forming units (CFUs) per gram, a threshold that approaches sterility [29]. In contrast, Canada permits a higher limit of up to 1000 CFUs per gram. Furthermore, the European and United States Pharmacopoeias have set strict microbiological safety standards for inhalable cannabis products, requiring the complete absence of pathogens such as *Staphylococcus aureus*, *Pseudomonas aeruginosa*, and bile-tolerant Gram-negative bacteria such as *Escherichia coli* [19,30]. Notably, in the United States, the standards for both recreational and medical cannabis are inconsistent across states [31]. Compared to this regulatory patchwork, microbial safety standards for hemp flower are even less consistent, often lacking clear oversight altogether, leaving hemp flower largely unregulated with respect to microbial load [24]. Furthermore, knowledge gaps remain regarding the impact of post-harvest treatments on flower quality and microbial load reduction, underscoring the need for studies to support evidence-based policymaking and standard-setting.

Given that maintaining sterile conditions in cannabis cultivation is both challenging and costly, effective post-harvest decontamination remains essential for consistently meeting safety requirements [32]. Post-harvest treatment methods, such as electron beam (EB), cold plasma, and gamma irradiation, have been thoroughly reviewed for their effectiveness in reducing fungal contaminants in medical cannabis [33,34,35,36]. Unlike gamma irradiation, EB technologies deliver ionizing radiation without relying on radioactive sources [37], making it a safer and more accepted technology within the industry [32]. While research has shown that EB irradiation can effectively sterilize cannabis flower, less is known about its impact on long-term storability, chemical integrity, and microbiological stability post-treatment [36]. This method, widely used in food safety, effectively sterilizes food ingredients and extends product shelf life [38], is regulated in the European Union, the United States, Australia, and Canada [37].

In addition to microbial safety, previous work has shown that the phytochemical composition of cannabis flowers changes during long-term storage. Cannabinoids such as Δ9-tetrahydrocannabinolic acid (THCA) and cannabidiolic acid (CBDA) undergo slow decarboxylation to their neutral forms, while THC can oxidize to cannabinol (CBN) under exposure to heat, light, or oxygen, whereas CBD is comparatively stable [7,30,39,40]. Terpenes are more vulnerable due to their volatility and susceptibility to oxidation; monoterpenes such as myrcene and limonene typically degrade more rapidly than sesquiterpenes, leading to measurable losses in aroma and quality over time [30,36,39]. Although packaging in sealed or vacuum-protected containers minimizes volatilization and oxygen exposure, several studies report significant declines in total terpene content over months of storage [30,39,41,42]. Recent work by Lumu et al. [40] demonstrated that hemp flowers stored for six months at different moisture levels retained higher total cannabinoid content when stored at lower moisture, while CBD and Δ9-THC levels remained statistically unchanged; however, elevated storage temperatures and light exposure accelerated cannabinoid decarboxylation [40]. These chemical dynamics are increasingly recognized as critical for product quality and consumer experience, yet systematic data on the combined effects of irradiation and storage on hemp flower phytochemistry remain limited.

This study evaluated the impact of EB irradiation and storage time on cannabinoid and terpene profiles, as well as microbial loads in hemp inflorescences of 2 cultivars over a 12-week storage period. Specifically, this research assesses the suitability of EB irradiation for sterilization, focusing on its effectiveness in preserving the chemical stability and microbial safety of hemp flowers during extended storage. Moreover, the study provides insights into the stability of hemp-specific secondary metabolites during storage and shows that microbial load decreases over time under the tested storage conditions. These findings highlight a potential avenue for further research into consumer safety standards.

## 2. Results and Discussion

### 2.1. Microbial Counting

Table 1 summarizes the mean and standard error (SE) of microbial counts (CFU/g) for Total Aerobic Microbial Count (TAMC) and Total Yeasts and Molds Count (TYMC) across storage durations (Week 0 to Week 12) and irradiation treatments. The data are presented separately for each of the two hemp cultivars studied (A and B).

#### 2.1.1. EB Irradiation

EB irradiation effectively reduced TAMC and TYMC in both cultivars, with irradiated samples consistently exhibiting microbial levels below the limit of detection (<100 CFU/g) across all storage timepoints (Table 1).

These results are in agreement with previous studies in which EB irradiation significantly reduced TYMC levels by around 5-log-fold in commercial medical cannabis inflorescences [36]. They are also consistent with findings from studies on maize seeds [43] and dried red pepper powder [44], where EB irradiation effectively reduced the initial fungal microbial load. Irradiated samples representing both cultivars showed microbial counts below the limit of detection (<100 ± 0 CFU/g) with no microbial recovery during storage, indicating sustained suppression of viability. The uniformity of microbial reduction underscores the ability of EB irradiation to reliably induce lethal damage to microbes, irrespective of initial contamination levels or storage duration, with no recovery observed over 12 weeks.

#### 2.1.2. Storage Time

Non-irradiated samples of both cultivars exhibited progressive declines in TAMC and TYMC over 12 weeks of storage, though with distinct patterns. For cultivar B, the TAMC decreased steadily from 20,728 CFU/g at Week 0 to <LOQ (100 CFU/g) by Week 12, while the TYMC declined from 606,667 CFU/g to 37,100 CFU/g over the same period. In contrast, cultivar A showed a sharp initial decline in TAMC (301,961 CFU/g at Week 0 to 21,274 CFU/g at Week 8), with fluctuations at later time points. TYMC in cultivar A decreased steadily from 4,311,111 CFU/g (Week 0) to 632,222 CFU/g (Week 12), but residual counts remained substantially higher than in cultivar B (17-fold higher for TYMC at Week 12). Microbial populations (CFU/g) in dried plant materials experience decline during storage due to the stress conditions created by the drying process. Bacteria are confronted with stress environments such as high or low temperature, higher osmolarity, and acidic pH [45]. While spore cells may remain viable for months after drying, the overall microbial load decreases over time as vegetative cells are more susceptible to the low water activity conditions than bacterial spores or certain resistant fungi, leading to a gradual reduction in total viable counts during storage. This natural die-off occurs due to cellular damage from desiccation stress, oxidative damage, and the inability to maintain metabolic processes at low moisture levels.

Cultivar A exhibited markedly higher initial microbial loads compared to cultivar B, with the Week 0 TAMC and TYMC levels 15-fold and 7-fold greater, respectively. This disparity likely contributed to higher variability in non-irradiated samples of cultivar A.

#### 2.1.3. Two-Way ANOVA Results for TAMC and TYMC

The two-way ANOVA results for TAMC and TYMC in the cannabis cultivars A and B are presented in Table 2.

Significant effects of EB irradiation treatment and storage time and their interaction were observed for all variables, with the exception of TAMC in Cultivar A. In Cultivar A, TYMC was strongly influenced by both irradiation treatment (*p* < 0.001) and storage time (*p* < 0.001). This indicates that EB irradiation effectively reduced fungal contamination while storage time contributed to a gradual decline in fungal counts, possibly due to natural decay. The significant interaction between irradiation treatment and storage time for TYMC (*p* < 0.001) further emphasizes the combined role of these factors in controlling fungal contamination over time. Effects of EB irradiation treatment and storage time and their interaction on TAMC were non-significant (*p* > 0.05).

In Cultivar B, both TAMC and TYMC were significantly affected by irradiation treatment (TAMC: *p* < 0.01; TYMC: *p* < 0.001) and storage time (TAMC: *p* < 0.01; TYMC: *p* < 0.001). This suggests that microbial populations in this cultivar were more uniformly responsive to both irradiation and the natural effects of storage time. The interaction between EB treatment and storage time was also significant for both parameters (TAMC: *p* < 0.01; TYMC: *p* < 0.001), indicating a synergistic effect of these factors in reducing microbial loads. These findings highlight the efficacy of EB irradiation in reducing microbial contamination, especially fungal growth (TYMC) which was significantly reduced in both varieties, although its effect on aerobic bacteria (TAMC) varied by cultivar. Together, these results underline the importance of post-harvest treatments like EB irradiation for ensuring microbial safety in cannabis products. The influence of cultivar on TAMC may warrant further investigation, as differences in secondary metabolites, such as terpenes and cannabinoids with known antimicrobial properties, could contribute to variation [46].

### 2.2. Mycotoxins

Mycotoxins were not detected in any of the samples from either cannabis cultivar at the start (Week 0) and end (Week 12) of the study, regardless of whether they received EB irradiation. Consequently, there were no observed effects of EB treatment or storage time on mycotoxin levels, nor any relationship between mycotoxin and TYMC or TAMC, suggesting that the microbes growing on these cultivars did not produce detectable mycotoxins.

### 2.3. Cannabinoid Content

**Figure 1:**
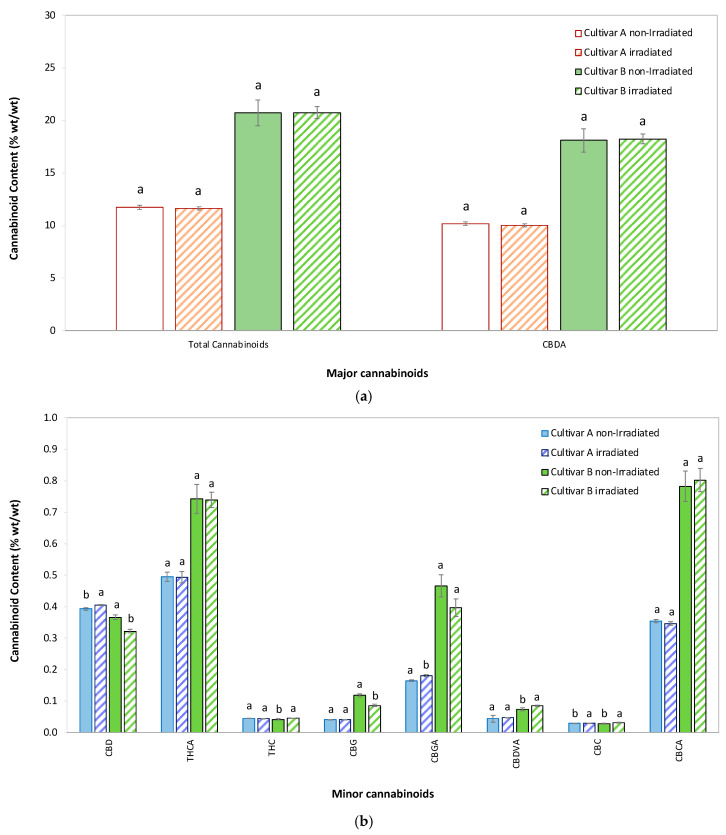
Mean ± SD of total and individual cannabinoid content in hemp flowers of cultivar A and cultivar B. (**a**) Comparison of major (>1% wt/wt) and (**b**) minor (0.01–1% wt/wt) cannabinoids after EB irradiation versus non-irradiated controls. CBC, cannabichromene; CBCA, cannabichromene acid; CBD, cannabidiol; CBDA, cannabidiolic acid; CBDVA, cannabidivarinic acid; CBG, cannabigerol; CBGA, cannabigerolic acid; EB, electron beam; SD, standard deviation; THC, Δ9-tetrahydrocannabinol; THCA, Δ9-tetrahydrocannabinolic acid. For each cannabinoid, identical lowercase letters above paired bars indicate no statistically significant difference between EB-irradiated and control samples (*p* > 0.05) (*n* = 3 biological replicates). Statistical analysis: One-way ANOVA (Welch’s test) followed by post hoc testing with Tukey’s HSD (for equal variances) to maximize sensitivity or Games-Howell (for unequal variances), based on Levene’s test results.

The mean ± standard deviation (SD) for major (>1% wt/wt) and minor (0.01–1% wt/wt) cannabinoids in flowers of both cultivars at Week 0 are presented in Figure 1a,b, respectively.

Total cannabinoid content remained largely unaffected by the EB irradiation in both cultivars, with cultivar B exhibiting a significantly higher total cannabinoid content (~20% wt/wt) compared to cultivar A (~12% wt/wt) across both EB-irradiated and non-irradiated samples. Cannabidiolic acid (CBDA) was the predominant cannabinoid in both cultivars, constituting the bulk of the total cannabinoid content. Levels of CBDA showed minimal variation between irradiated and non-irradiated samples, indicating that EB irradiation had no significant impact on its concentration. Similarly, minor cannabinoids such as THC, THCA, cannabigerol (CBG), cannabigerolic acid (CBGA), and others were present in much smaller quantities and demonstrated negligible changes due to irradiation. Overall, the data suggest that the cannabinoid profile of cannabis flowers is highly stable with EB irradiation, with no substantial reductions observed for either major or minor cannabinoids in either cultivar. These findings highlight the resilience of cannabinoids to post-harvest irradiation processing.

Supplementary Table S1 details the effects of E-beam (EB) irradiation and 12-week storage on total cannabinoid contents (sum of acidic + neutral forms) in the two high-CBD cannabis cultivars. Critically, neither irradiation nor storage significantly altered total cannabinoids or CBD in either cultivar, confirming the stability of these primary quality traits under experimental conditions.

In Cultivar A, EB irradiation significantly affected minor cannabinoids: total THC (*p* < 0.05) and total CBC (*p* < 0.01). Storage reduced total THC, CBG, and CBC (*p* < 0.001) and CBDV (*p* < 0.05), while irradiation × storage interactions modified degradation kinetics for THC (*p* < 0.05), CBG (*p* < 0.01), and CBC (*p* < 0.01). The significant effects of E-beam irradiation and storage on total THC, CBC, and CBG—but not total cannabinoids or CBD—reflect inherent structural vulnerabilities in these minor compounds. THC’s susceptibility stems from its oxidizable phenolic hydroxyl and strained cyclohexene double bond. CBC’s instability arises from its conjugated diene system within the chromene ring. CBG’s heightened sensitivity is primarily driven by its linear, electron-rich geranyl chain, featuring reactive allylic positions and a vulnerable terminal isoprene unit prone to oxidation and electrophilic attack, combined with the inherent reactivity of its resorcinol core. Conversely, CBD’s stabilized resorcinol architecture within its pyran ring and the absence of highly reactive alkenes or exposed allylic systems in its core structure explain its resilience. Critically, the stability of total cannabinoids and CBD—the dominant commercial traits—validates irradiation as a decontamination method for high-CBD cultivars, despite cultivar-specific fluctuations in minor analogs.

Cultivar B exhibited greater resilience: irradiation alone caused no significant changes in any cannabinoid. Storage reduced only total CBG (*p* < 0.001) and CBC (*p* < 0.05), with a single interaction effect for CBG (*p* < 0.01). The stability of total cannabinoids and CBD—the dominant commercial traits—supports EB irradiation as a viable decontamination method for high-CBD cultivars. While minor cannabinoids (e.g., THC, CBC, CBG) showed cultivar-dependent degradation during storage, these changes do not compromise core product integrity. Cultivar A’s greater sensitivity in minor compounds highlights genetic influences on stability, urging cultivar-specific validation. Nevertheless, the preservation of major cannabinoids underscores irradiation’s compatibility with industry requirements for shelf-stable, compliant products.

### 2.4. Terpene Content

**Figure 2:**
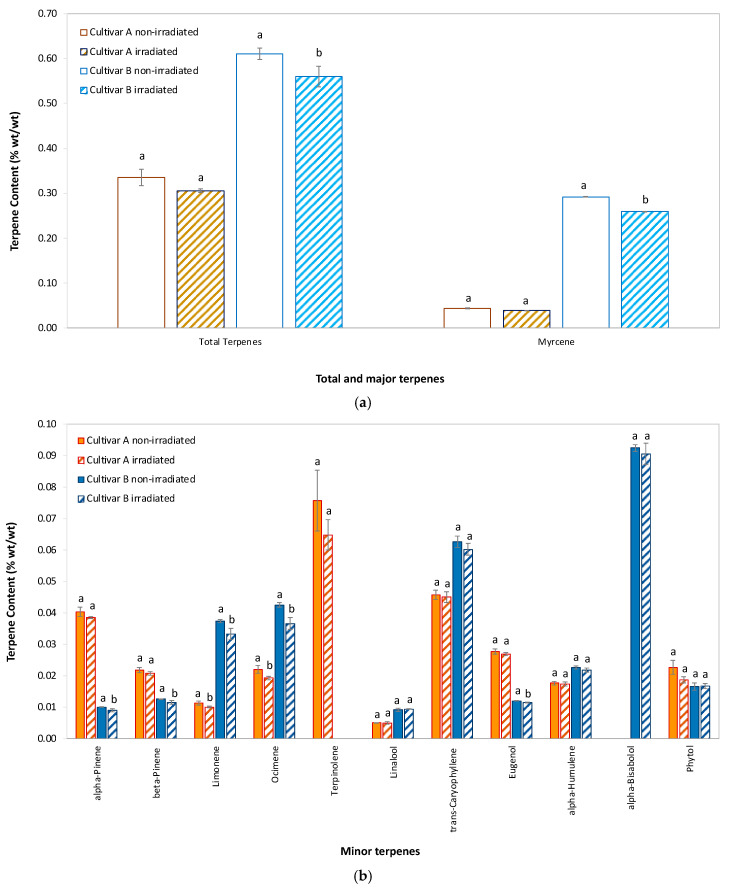
Mean ± SD of total and individual terpene content in hemp flowers of cultivar A and cultivar B. Comparison of (**a**) total and major terpenes (>0.1% wt/wt) and (**b**) minor terpenes (0.01–0.1% wt/wt) after EB irradiation versus non-irradiated controls at W0. For each terpene, identical lowercase letters above paired bars indicate no statistically significant difference between EB-irradiated and control samples (*p* > 0.05) (*n* = 3 biological replicates). Statistical analysis: One-way ANOVA (Welch’s test) followed by post hoc testing with Tukey’s HSD (for equal variances) to maximize sensitivity or Games-Howell (for unequal variances)**,** based on Levene’s test results. Note: alpha-bisabolol in Cultivar A and terpinolene in Cultivar B were below LOQ (0.01% wt/wt), resp., thus are not displayed.

Figure 2 shows the mean ± SD for major (>0.1% wt/wt) (Figure 2a) and minor (0.01–0.1% wt/wt) (Figure 2b) terpenes in hemp flowers from cultivar A and cultivar B immediately following EB irradiation at the start of the study (Week 0), compared with non-irradiated controls. Non-irradiated cultivar B samples had almost twice the total terpene content of non-irradiated cultivar A samples (0.61% vs. 0.33%), with notably higher levels of myrcene (0.29% vs. 0.04%) and alpha-bisabolol (0.09% vs. <0.01% [i.e., below the limit of detection]), while cultivar A had higher terpinolene content than cultivar B (0.09% vs. <0.01%). The relative reduction in total terpene content with irradiation was minor at 8.6% and 8.3% for cultivar A and cultivar B, respectively. Myrcene, the most abundant individual terpene (up to 0.29%), showed a noticeable reduction in irradiated samples, particularly for cultivar B. Other major terpenes, such as limonene, terpinolene, and ocimene, also displayed decreased levels following irradiation. Alpha-pinene, beta-pinene, trans-caryophyllene, and linalool were less affected, though slight reductions were observed in irradiated samples. Cultivar B samples maintained higher baseline levels of these terpenes compared with cultivar A samples, regardless of whether they received irradiation. Minor constituents such as eugenol (phenylpropanoid), alpha-humulene, alpha-bisabolol, and phytol (diterpenoid alcohol) exhibited minimal differences between irradiated/non-irradiated samples, with both cultivars showing low levels overall.

These findings suggest that EB irradiation primarily causes the rapid loss of monoterpenes shortly after treatment with minimal effect on more stable sesquiterpenes, indicating that changes to terpene content from EB irradiation are largely limited to the immediate post-treatment period.

Supplementary Table S2 (two-way ANOVA for total and individual terpene contents) reveals fundamental differences in how Cultivars A and B respond to E-beam irradiation and storage. While irradiation significantly affected multiple terpenes in Cultivar A (*p* < 0.05 to *p* < 0.01), it showed no effect on total terpenes. In stark contrast, Cultivar B exhibited profound irradiation sensitivity, with significant effects on total terpenes (*p* < 0.001) and nearly all individual compounds. Storage time emerged as the dominant stressor, explaining the greatest proportion of variance (highest *F*-values) across both cultivars and causing significant effects (*p* < 0.001) on nearly all terpenes. The consistently larger *F*-values for storage versus irradiation and their interaction indicate it drives the majority of terpene variability.

Notably, both factors affected the cultivars differently. Cultivar B’s higher sensitivity to irradiation coincided with substantially higher initial terpene concentrations (0.65% vs. 0.37% wt/wt in Cultivar A) and distinct composition—particularly its myrcene dominance (45–48% of total terpenes vs. 11–13% in cultivar A). This compositional divergence suggests inherent biochemical differences: higher terpene load and abundant volatile monoterpenes (e.g., myrcene, limonene) likely increase susceptibility to EB irradiation as well as oxidative degradation pathways.

The minimal EB irradiation × Storage interactions in Cultivar B (vs. widespread interactions in cultivar A) further highlight fundamental metabolic differences. Cultivar A’s significant interactions indicate irradiation modified storage degradation kinetics—potentially through residual oxidative effects. Cultivar B’s limited interactions suggest its terpenes experienced immediate, irreversible damage from irradiation, leaving little substrate for time-dependent interactions.

In conclusion, the stark contrast in terpene stability between cultivars aligns primarily with compositional differences: Cultivar B’s higher initial terpene content (0.65% vs. 0.37% in Cultivar A) and monoterpene-dominant profile (e.g., myrcene: 45–48% vs. 11–13%) likely amplified its vulnerability. Volatile monoterpenes (e.g., myrcene, limonene) are inherently prone to degradation under EB irradiation and storage, explaining B’s significant effects. Cultivar A’s stability may arise from its lower terpene load and distinct profile, though biochemical factors (e.g., antioxidants) could potentially contribute. Critically, microbial burden (higher in A) did not predict stability, underscoring that composition—not contamination—drives degradation risk. For high-terpene cultivars, irradiation protocols should be optimized to preserve aroma-critical compounds.

### 2.5. EB Irradiation Effect on Monoterpene and Sesquiterpene Content at Week 0

**Figure 3:**
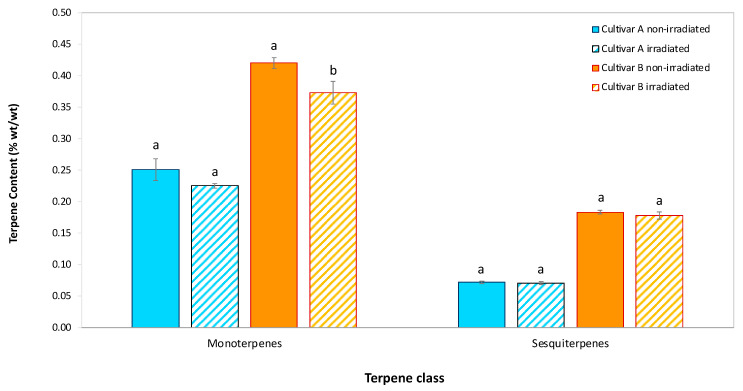
Mean ± SD for monoterpene and sesquiterpene content (wt/wt) in hemp flowers of cultivar A and cultivar B after EB irradiation versus non-irradiated controls at Week 0. EB, electron beam; SD, standard deviation. For each terpene class, identical lowercase letters above paired bars indicate no statistically significant difference between EB-irradiated and control samples (*p* > 0.05) (*n* = 3 biological replicates). Statistical analysis: One-way ANOVA (Welch’s test) followed by Tukey’s HSD post hoc testing.

The immediate effect (Week 0) of EB irradiation on the content of monoterpenes and sesquiterpenes in the two hemp cultivars is presented in Figure 3.

The data reveal that EB irradiation results in a reduction in terpene content, with monoterpenes being more significantly affected than sesquiterpenes. In cultivar A, the monoterpene content decreased from 0.25% in non-irradiated samples to 0.23% in irradiated samples, indicating a relative reduction of 10.3%. Similarly, in cultivar B, the monoterpene content dropped from 0.42% in non-irradiated samples to 0.37% in irradiated samples, representing an 11.2% relative decrease. For sesquiterpenes, the reduction in content was much smaller. In cultivar A, sesquiterpene content decreased by 2.2%, while in cultivar B, the reduction was around 2.7%. This greater reduction in monoterpenes is likely attributable to their higher volatility. The lower volatility of sesquiterpenes, which consist of three isoprene units, explains their relatively minor reduction compared to monoterpenes. These results suggest that the immediate impact of EB irradiation is primarily on the evaporation of monoterpenes due to their higher volatility. The minimal impact on sesquiterpenes underscores their greater stability under similar conditions. This suggests that the EB treatment’s effect on terpene content is limited to the initial period following irradiation.

### 2.6. Effect of EB Irradiation × Storage Time on Monoterpene-to-Sesquiterpene Ratio

**Figure 4:**
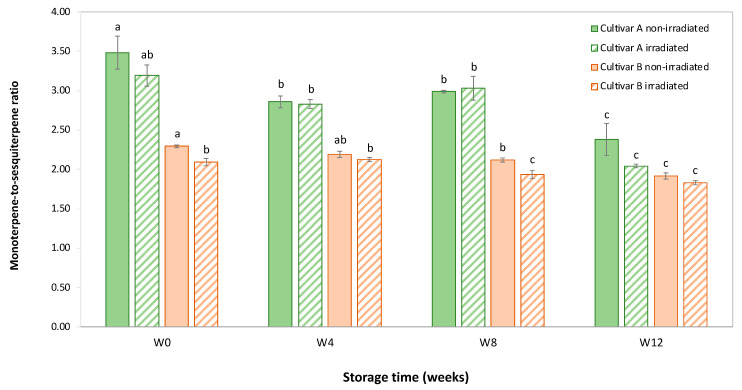
Mean ± SD for the monoterpene-to-sesquiterpene ratio in hemp flowers of cultivar A and cultivar B after EB irradiation versus non-irradiated controls at W0, W4, W8, and W12. EB, electron beam; SD, standard deviation; W, week. Within each cultivar, identical lowercase letters which includes irradiated and non-irradiated control across different storage times (W0–W12) indicate no statistically significant difference (*p* > 0.05) (*n* = 3 biological replicates; two-way ANOVA (factors: Weeks × EB Irradiation Treatment) with Bonferroni post hoc testing).

EB irradiation caused an immediate reduction in the monoterpene-to-sesquiterpene ratio in both hemp cultivars (Figure 4).

At Week 0, the monoterpene-to-sesquiterpene ratio in cultivar A was 3.48 in non-irradiated samples and 3.19 in irradiated samples. Similarly, in cultivar B, the respective ratios were 2.29 and 2.09. This immediate reduction is consistent with the higher volatility of monoterpenes, which are more susceptible to degradation or evaporation following EB irradiation. Over time, both cultivars showed a gradual decline in the ratio for both irradiated and non-irradiated samples. In cultivar A, the ratio in non-irradiated samples decreased from 3.48 at Week 0 to 2.38 at Week 12, while the ratio in irradiated samples declined from 3.19 to 2.04 over the same period. In cultivar B, the ratio dropped from 2.29 to 1.92 in non-irradiated samples and from 2.09 to 1.83 in irradiated samples from Week 0 to Week 12. Interestingly, by Week 12, the difference in the monoterpene-to-sesquiterpene ratio between irradiated and non-irradiated samples diminished, suggesting that the initial effects of EB irradiation on terpene volatility even out over time. This convergence implies that while EB irradiation accelerates the initial loss of monoterpenes, the long-term degradation rates of both monoterpenes and sesquiterpenes become similar as the more volatile monoterpenes stabilize or reach equilibrium.

### 2.7. Effects of EB Irradiation × Storage Time on Relative Terpene Abundance

**Figure 5:**
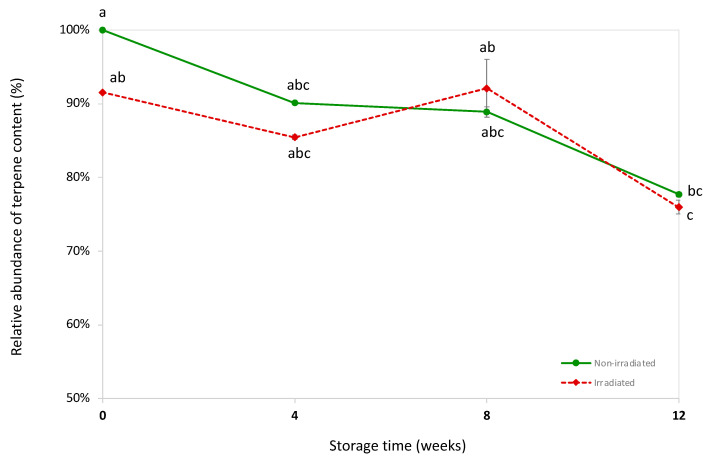
Mean ± SD for the relative abundance of total terpene content in hemp flowers of cultivars A and B after EB irradiation versus non-irradiated controls at 0, 4, 8, and 12 weeks of storage. The data are expressed as the relative percentage of the initial total terpene content in the non-irradiated control flowers at Week 0 (set at 100%). Identical lowercase letters, which includes irradiated and non-irradiated control across different storage weeks 0–12, indicate no statistically significant difference (*p* > 0.05) (*n* = 3 biological replicates; two-way ANOVA (factors: Weeks × EB Irradiation Treatment) with Bonferroni post hoc testing). Note: corresponding results and charts for each individual cultivar are provided in Supplementary Table S3.

Figure 5 illustrates the change in total terpene content across both cultivars as a function of both EB irradiation and storage time. The data are expressed as relative abundance of terpenes, which was calculated as a percentage of the initial terpene content (Week 0) reported in non-irradiated (control) samples (set at 100%).

The immediate 8.4% reduction following EB irradiation likely results from direct radiolytic effects, where high-energy electrons induce bond scission in terpene molecules, particularly affecting volatile monoterpenes through homolytic cleavage and formation of reactive intermediates. Over the 12-week storage period, both the non-irradiated and EB-irradiated samples exhibited a gradual decline in terpene content. This storage-related decrease could involve oxidative degradation, where residual oxygen reacts with terpene double bonds through autoxidation, although the samples were packed and stored in vacuum-sealed bags, which should minimize oxygen exposure and volatilization. Other possible degradation pathways may also contribute, such as hydrolysis or elimination reactions, even in dried samples. A notable exception occurred at Week 8 for the EB-irradiated samples, where an apparent increase was observed. However, this deviation is most likely attributable to the relatively large SD for that time point rather than a genuine increase in terpene content. By the end of the 12-week storage period, both non-irradiated and irradiated samples showed similar overall terpene losses (22.3% vs. 24.0%, respectively), indicating that EB irradiation does not substantially alter the chemical stability of the remaining terpene fraction or generate catalytic species that would accelerate subsequent degradation processes. The preservation of sesquiterpenes in both treatments supports this conclusion, as their lower volatility and reduced oxidation susceptibility contribute to overall profile stability during storage. These results suggest that while EB irradiation causes an initial reduction in some terpene levels, storage time exerts a more pronounced overall effect. The relatively small impact of EB irradiation on terpene stability indicates that this sterilization method is compatible with preserving terpene profiles.

## 3. Materials and Methods

Female plants from hemp cultivars A and B were cultivated under controlled glasshouse conditions in Weiningen, Switzerland, during the 2024 spring-summer season. Cuttings from genetically identical mother plants were rooted in a climate-controlled environment for 14 days at 26 °C with 85% relative humidity and an 18 h photoperiod (18 h light/6 h dark). Light intensity was gradually increased from 100 to 300 µmol·m^−2^·s^−1^ (Photosynthetic Photon Flux Density) during this period. Once rooted, the cuttings were transplanted into 5 L pots filled with coco-peat substrate and moved to a vegetative room within the glasshouse. During the vegetative phase, the plants were maintained under an 18 h photoperiod, with temperatures averaging 28 °C in the daytime and 25 °C at night; relative humidity ranged between 70 and 85%. After 7 days, plants were transferred to flowering conditions for approximately 7 weeks, with the photoperiod adjusted to 12 h light/12 h dark to induce flowering. During the flowering phase, daytime and nighttime temperatures averaged 27 °C and 23 °C, respectively, and relative humidity was gradually decreased from 75% to 60%. Supplemental CO_2_ was provided based on external radiation levels. Plants were irrigated using an ebb-and-flow system with a nutrient solution, where the electrical conductivity was progressively reduced from 1.8 dS·m^−1^ to 1.0 dS·m^−1^ throughout the flowering period. Supplemental lighting was used during both the vegetative and flowering phases when outdoor radiation was insufficient for optimal growth. Blackout screens were employed to regulate the photoperiod.

At the end of the flowering phase, the plants were manually harvested, with flowers carefully separated from the stems, trimmed, and freeze-dried. Water activity (a_w_) was not measured during storage; however, the flowers were dried to typical commercial moisture levels prior to packaging. Two kilograms of dried hemp flowers were then placed inside Mylar bags [3.5 mil (89 µm) laminate of polyethylene terephthalate, aluminum foil, and polyethylene (PET/foil/PE)]. These multilayer bags are designed to provide a strong barrier against moisture and oxygen transmission and are widely used for long-term storage of dried botanicals. Irradiation was carried out by an industrial partner (Ionisos EB Services AG, Däniken, Switzerland) as a tolling service. The processing conditions, using a high-energy (9.8 million electron Volts, 28 kW) linear accelerator-based electron beam system were: 120 cm scan width, single-pass configuration, conveyor speed of 3 m/min, and beam current of 1.86 mA. Detailed dose mapping data were not available to the authors. The speed of the conveyor belt was adjusted to deliver ca. 5 kGy dose to the material from the accelerator. After treatment, the plant material was packed into vacuum-sealed bags, each containing approximately 50 g of flower material. Packing was carried out in a sterile environment to prevent additional contamination. The bags were then placed along similarly packed non-irradiated flower material (controls) in a storage room maintained at 17–19 °C with a relative humidity of around 50%. Sampling followed a fixed schedule: immediately post-packaging (Week 0, baseline) and at Weeks 4, 8, and 12 of storage. At each time point, new bags (*n* = 3 per cultivar and treatment) were retrieved and analyzed for cannabinoids, terpenes, and microbiology as described. Table 3 provides a comprehensive summary of the analytical and microbiological tests performed on the samples. All measurements and tests were conducted in triplicate to ensure the reliability and reproducibility of the results.

### 3.1. Determination of Cannabinoids and Terpenes

Cannabinoids were quantified following the protocol outlined by Giese et al. [47]. Hemp flowers were manually ground, and the homogenized biomass was weighed to approximately 200 mg. The material was transferred to a 15 mL polypropylene centrifuge tube (BD, Franklin Lakes, NJ, USA), and 10 mL of ethanol was added. The samples were briefly vortexed, followed by sonication for 10 min at room temperature. The supernatant was filtered through a 0.2 μm polytetrafluoroethylene syringe filter, and the filtrate was dispensed in 1–2 mL aliquots, which were then diluted 20-fold in ethanol before being transferred into high-performance liquid chromatography (HPLC) and gas chromatography (GC) glass vials, which were stored at −20 °C until further analysis. Acidic and neutral forms of cannabinoids were detected, identified, and quantified using an Agilent 1290 Infinity II UPLC-DAD system (Agilent Technologies, Inc., Santa Clara, CA, USA). The mobile phases consisted of two HPLC-grade solutions: solution A (0.1% *v*/*v* aqueous formic acid) and solution B (methanol with 0.05% *v*/*v* formic acid). A Kinetex 1.7 μm EVO C18 100 Å column (100 mm × 2.1 mm; Phenomenex Inc., Torrance, CA, USA) solid phase was utilized. Compound peaks were detected for 230 nm and 310 nm. Cannabinoid determinations were run in triplicate. The following cannabinoids were quantified: THC, THCA, CBD, CBDA, CBG, CBGA, CBDV, CBDVA, cannabinol (CBN), CBC, and CBCA. Analytes that were below the limit of quantification (LOQ) were not included in the Results.

The following formulas were used to calculate the total cannabinoid content in both neutral and acidic forms:

Total THC = THC + THCA × 0.877

Total CBD = CBD + CBDA × 0.877

Total CBG = CBG + CBGA × 0.878

Total CBDV = CBDV + CBDVA × 0.867

Total CBC = CBC + CBCA × 0.877

The conversion factors used (e.g., 0.877 for THCA and CBDA) account for the mass lost during decarboxylation (i.e., release of CO_2_) when acidic cannabinoids are converted to their neutral forms. Decarboxylation rates were calculated as the percentage conversion of the acidic cannabinoid forms into their neutral counterparts on a weight basis, to evaluate the effects of cultivar, storage time, and irradiation treatment on this process.

Terpene analysis was carried out using an Agilent 6890 GC-FID system equipped with a split/splitless injector; the method was adapted from the application notes of the manufacturer. Samples were prepared by diluting the cannabis extract described above tenfold in ethanol. A liquid injection of 1 µL was performed in split mode, with a split ratio optimized to avoid detector saturation. Separation was achieved on an Agilent DB-5ms column (30 m × 0.25 mm × 0.25 µm), with helium as the carrier gas at a constant flow rate of 1.0 mL/min. The oven temperature program began at 50 °C (held for 1 min), increased at 10 °C/min to 150 °C, then at 5 °C/min to 250 °C, with a final hold of 5 min. The injector and detector temperatures were maintained at 250 °C and 280 °C, respectively. A series of terpene standards was used for calibration, and quantification was achieved by comparing peak areas of target compounds to those of external standards. The terpenes were identified by comparing the retention times of the chromatographic peaks with those of reference standards obtained from Sigma-Aldrich run under the same conditions.

### 3.2. Microbiological Tests

All microbiological tests were carried out by ISO-certified third-party laboratories following standard procedures. TYMC and TAMC were conducted in triplicate following the guidelines outlined in the *European Pharmacopoeia* [48,49]. CompactDry plates (Shimadzu, Kyoto, Japan) were utilized for both TYMC and TAMC assessments. Peptone buffer, prepared according to the manufacturer’s instructions, served as the nutrient medium. The CompactDry plates were prepared and handled according to the manufacturer’s instructions. Hemp flower material was suspended in peptone buffer, and the resulting suspension was subjected to serial dilutions to obtain suitable concentrations for microbial enumeration. To distinguish bacterial colonies from yeast and mold, antibiotics were added during the inoculation process. Diluted samples were inoculated onto the CompactDry plates and incubated at specified temperatures to promote selective growth (TAMC, 35 °C; TYMC, 28 °C). After incubation, microbial colonies were visually counted, and the results were expressed as CFU per gram of hemp flower material. Mycotoxins were detected using HPLC coupled with fluorescence detection, following the procedures outlined in the *European Pharmacopoeia* [50]. *Salmonella*, and *E. coli* were detected as described in the guidelines outlined in the *European Pharmacopoeia* [49].

### 3.3. Statistical Analysis

Cannabinoid and terpene data were analyzed using a three-way ANOVA with cultivar (A vs. B), irradiation treatment (irradiated vs. non-irradiated), and storage time as major factors.

## 4. Conclusions

EB irradiation was highly effective in reducing microbial loads in hemp, achieving levels far below current regulatory thresholds for medical cannabis immediately after treatment. Notably, non-irradiated samples also exhibited a significant decrease in microbial loads over the 12-week storage period, though they remained higher than those in the irradiated samples overall. As water activity (aw) was not monitored during storage, we cannot assess the observed microbial declines in the context of moisture conditions; future work should incorporate aw measurements alongside microbial counts to clarify this relationship. The chemical composition of quality-critical compounds, including cannabinoids and terpenes, was well-preserved, with minimal or no impact observed from EB irradiation treatment. In this study, CBD, THC, and other cannabinoids appeared unaffected by EB irradiation or subsequent storage, with no measurable changes observed in either irradiated or non-irradiated samples. EB irradiation immediately reduces the monoterpene-to-sesquiterpene ratio, likely because of the greater volatility of monoterpenes. However, there is no indication that the initial EB irradiation has any lasting impact on the natural degradation of terpenes over time. Regardless of whether samples received irradiation, increased storage time led to a reduction in total terpene content, underscoring the need for optimizing storage conditions and preservation methods to maintain hemp flower quality. Collectively, these findings support EB irradiation as a robust decontamination method and highlight storage duration as a promising post-harvest intervention, which warrants further exploration for optimizing microbial reduction.

## Figures and Tables

**Table 1:** Mean ± SE for TAMC and TYMC in hemp cultivars A and B, without and with EB irradiation treatment, by storage time.

Treatment	Storage Time	TAMC (CFU/g)	TYMC (CFU/g)
*Cultivar A*			
Non-Irradiated	Week 0	301,961 ± 264,606	4,311,111 ± 997,791
	Week 4	104,480 ± 67,892	2,466,667 ± 195,316
	Week 8	21,274 ± 9605	1,144,444 ± 48,432
	Week 12	68,204 ± 44,411	632,222 ± 68,430
Irradiated	Week 0	<100 ± 0	<100 ± 0
	Week 4	<100 ± 0	<100 ± 0
	Week 8	<100 ± 0	<100 ± 0
	Week 12	<100 ± 0	<100 ± 0
*Cultivar B*			
Non-Irradiated	Week 0	20,728 ± 8761	606,667 ± 104,775
	Week 4	3233 ± 1772	192,333 ± 88,556
	Week 8	767 ± 617	83,900 ± 33,744
	Week 12	<100 ± 0	37,100 ± 28,587
Irradiated	Week 0	<100 ± 0	<100 ± 0
	Week 4	<100 ± 0	<100 ± 0
	Week 8	<100 ± 0	<100 ± 0
	Week 12	<100 ± 0	<100 ± 0

Note: EB, electron beam; TAMC, Total Aerobic Microbial Count; TYMC, Total Combined Yeast and Mold Count; CFU/g, Colony-forming Units per gram.

**Table 2:** *F* Values of two-way ANOVA for the microbiological tests TAMC and TYMC in cultivars A and B in relation to EB irradiation treatment and storage time.

Factor	*F* Values			
	*Cultivar A*		*Cultivar B*	
	TAMC	TYMC	TAMC	TYMC
Irradiation Treatment	3.20	70.30 ***	7.06 **	40.65 ***
Storage Time	0.80	10.36 ***	5.05 **	12.98 ***
Irradiation Treatment * Storage Time	0.79	10.36 ***	4.47 **	12.93 ***

***, ** and * means statistically significant at *p* < 0.001, 0.01 and 0.05, resp.

**Table 3:** Overview of chemical analyses and microbiological tests performed on EB-treated and untreated hemp samples over the storage time (0–12 weeks).

Analysis	Description	Method	Biological Replicates	Analytical Replicates
Cannabinoids	Acidic and neutral forms of major cannabinoids	UPLC-DAD	3 bags/sampling date	3
Terpenes	Terpenes (LOQ > 0.01)	GC-FID	3 bags/sampling date	3
Microbiology	TYMC	Plate counting	3 bags/sampling date	3
Microbiology	TAMC	Plate counting	3 bags/sampling date	3
Microbiology	*Salmonella*, *Escherichia coli*	Plate counting	3 bags/sampling date	1
Mycotoxins	Aflatoxin B1, aflatoxin Σ (B1, B2, G1, G2), ochratoxin A	LC	3 bags/sampling date	3

Sampling dates correspond to 0, 4, 8, and 12 weeks of storage. GC-FID, gas chromatography-flame ionization detection; UPLC-DAD, ultra-performance liquid chromatography with diode-array detection; LC, liquid chromatography; LOQ, limit of quantification; TAMC, Total Aerobic Microbial Count; TYMC, Total Yeast and Mold Count.

## Data Availability

The data presented in this study are available upon request from the corresponding author.

## Supplementary Materials

The following supporting information can be downloaded at https://www.mdpi.com/article/10.3390/molecules30173601/s1: Supplementary Table S1: *F* values of two-way ANOVA for total cannabinoids, CBD, THC, CBG, CBDV, and CBC in cultivars A and B in relation to irradiation treatment and storage time; Supplementary Table S2: *F* values of two-way ANOVA for total and individual terpenes in cultivars A and B in relation to irradiation treatment and storage time; Supplementary Table S3: Mean ± SD for the relative abundance of total terpene content in hemp flowers of cultivars A and B after EB irradiation versus non-irradiated controls at 0, 4, 8, and 12 weeks of storage.
