# Supplementary material for: Impact of Electron Beam Treatment and Storage Duration on Microbial Stability and Phytochemical Integrity in Hemp Flowers
Source: Molecules. 2025 Sep 3;30(17):3601. doi: 10.3390/molecules30173601 (PMC12430370; doi:10.3390/molecules30173601)

molecules

MDPI

Supplementary Material

Supplementary Table S1. *F* values of two-way ANOVA for total cannabinoids, CBD, THC, CBG, CBDV, and CBC in cultivars A and B in relation to irradiation treatment and storage time.

Factor	*F* values					
	Total cannabinoids	Total CBD	Total THC	Total CBG	Total CBDV	Total CBC
Cultivar A						
Irradiation treatment	0.00	0.08	5.78*	0.11	2.39	10.82**
Storage time	0.85	0.40	18.04***	31.63***	4.6*	37.43***
Irradiation treatment × Storage time	0.38	0.53	3.66*	8.04**	2.1	8.95**
Cultivar B						
Irradiation treatment	1.88	2.01	2.51	1.2	0.12	0.1
Storage time	1.74	1.03	3.07	23.91***	2.35	4.2*
Irradiation treatment × Storage time	0.82	0.99	1.05	8.01**	1.26	0.6

Note: *$p<0.05$, **$p<0.01$, ***$p<0.001$. ANOVA, analysis of variance; CBC, cannabichromene; CBD, cannabidiol; CBDV, cannabidivarin; CBG, cannabigerol; THC, Δ9-tetrahydrocanna-binol.

molecules

MDPI

Supplementary Table S2. *F* values of two-way ANOVA for total and individual terpenes in cultivars A and B in relation to irradiation treatment and storage time.

Factors	*F* values											
	Total terpenes	Alpha-pinene	Beta-pinene	Myrcene	Limonene	Ocimene	Terpinolene	Linalool	Trans-caryophyllene	Eugenol	Alpha-humulene	Phytol
Cultivar A												
Irradiation treatment	0.11	13.73**	13.73*	0.87	1.49	0.00	5.81*	2.73	4.88*	2.60	5.64*	8.84**
Storage time	33.38***	38.57***	38.57***	24.74***	9.11**	19.84***	44.1***	17.96***	0.11	11.03***	0.15	13.79***
Irradiation treatment × Storage time	6.98**	10.06**	10.06**	11.7***	9.84**	7.26**	3.33*	3.06	5.18*	4.87*	5.78**	4.87*
Cultivar B												
Irradiation treatment	30.21***	6.42*	14.30**	35.68***	42.70***	68.67***	-	3.81	6.28*	59.56***	6.30*	0.22
Storage time	58.56***	30.55***	242.09***	72.68***	46.92***	79.06***	-	50.08***	40.39***	42.14***	48.78***	25.22***
Irradiation treatment × Storage time	1.50	1.38	2.31	1.85	1.13	1.83	-	4.81*	1.31	4.59*	1.94	6.03**

Note: *p<0.05, **p<0.01, ***p<0.001. ANOVA, analysis of variance.

Supplementary Table S3. Mean ± SD for the relative abundance of total terpene content in hemp flowers of cultivars A and B after EB irradiation versus non-irradiated controls at 0, 4, 8, and 12 weeks of storage.

**Cultivar A: Relative Total Terpenes (% w/w)**		
Weeks	Control	Irradiated
0	100%	91.4%
4	91.1%	85.9%
8	88.4%	97.6%
12	74.6%	77.3%

**Cultivar B: RelativeTotal Terpenes (% w/w)**		
Weeks	Control	Irradiated
0	100.0%	91.7%
4	89.1%	85.0%
8	89.4%	86.5%
12	80.8%	74.6%

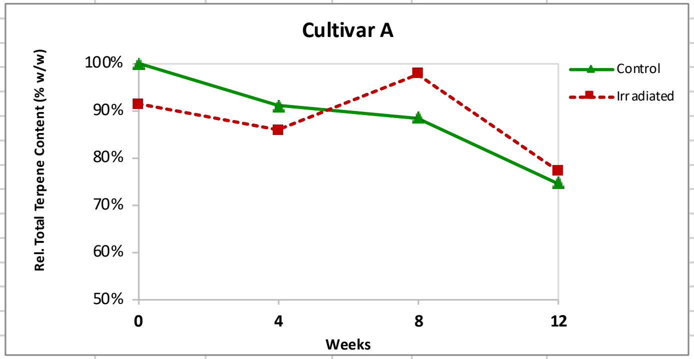

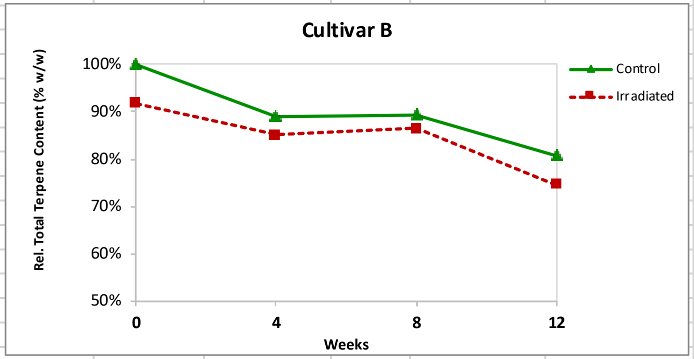

**Mean for Cultivars A and B:** Relative Total Terpenes (% w/w)				
Weeks	Control	Irradiated	SD Control	SD Irradiated
0	100.0%	91.6%	0.00	0.00
4	90.1%	85.4%	0.01	0.00
8	88.9%	92.1%	0.00	0.04
12	77.7%	76.0%	0.02	0.01

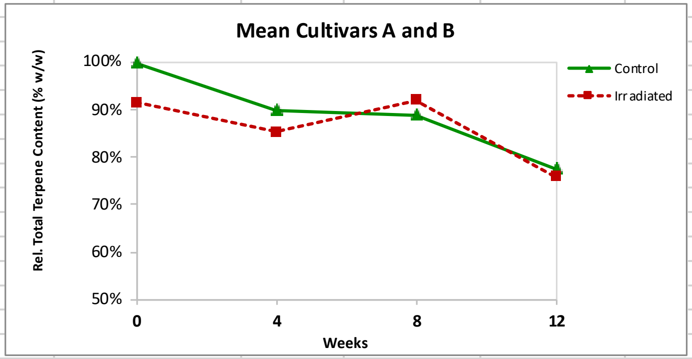

Supplement: Supplementary file 1 [file molecules-30-03601-s001.zip › molecules-3794499-supplementary.pdf]
